# Composition of Gut Microbiota Influences Resistance of Newly Hatched Chickens to *Salmonella* Enteritidis Infection

**DOI:** 10.3389/fmicb.2016.00957

**Published:** 2016-06-17

**Authors:** Karolina Varmuzova, Tereza Kubasova, Lenka Davidova-Gerzova, Frantisek Sisak, Hana Havlickova, Alena Sebkova, Marcela Faldynova, Ivan Rychlik

**Affiliations:** Veterinary Research Institute, BrnoCzech Republic

**Keywords:** chicken, microbiota, *Salmonella* Enteritidis, cecum, inflammation, competitive exclusion

## Abstract

Since poultry is a very common source of non-typhoid *Salmonella* for humans, different interventions aimed at decreasing the prevalence of *Salmonella* in chickens are understood as an effective measure for decreasing the incidence of human salmonellosis. One such intervention is the use of probiotic or competitive exclusion products. In this study we tested whether microbiota from donor hens of different age will equally protect chickens against *Salmonella* Enteritidis infection. Newly hatched chickens were therefore orally inoculated with cecal extracts from 1-, 3-, 16-, 28-, and 42-week-old donors and 7 days later, the chickens were infected with *S*. Enteritidis. The experiment was terminated 4 days later. In the second experiment, groups of newly hatched chickens were inoculated with cecal extracts of 35-week-old hens either on day 1 of life followed by *S*. Enteritidis infection on day 2 or were infected with *S*. Enteritidis infection on day 1 followed by therapeutic administration of the cecal extract on day 2 or were inoculated on day 1 of life with a mixture of the cecal extract and *S*. Enteritidis. This experiment was terminated when the chickens were 5 days old. Both *Salmonella* culture and chicken gene expression confirmed that inoculation of newly hatched chickens with microbiota from 3-week-old or older chickens protected them against *S*. Enteritidis challenge. On the other hand, microbiota from 1-week-old donors failed to protect chickens against *S*. Enteritidis challenge. Microbiota from 35-week-old hens protected chickens even 24 h after administration. However, simultaneous or therapeutic microbiota administration failed to protect chickens against *S*. Enteritidis infection. Gut microbiota can be used as a preventive measure against *S*. Enteritidis infection but its composition and early administration is critical for its efficacy.

## Introduction

Non-typhoid *Salmonella enterica* serovars are among the most common causative agents of food-borne diseases worldwide. Since poultry belongs among the most frequent reservoirs of *Salmonella* for humans, different interventions, such as strict hygiene measures or vaccination are applied to decrease *Salmonella* prevalence in chickens. However, further improvements in hygiene measures are costly. Current vaccination regimes effectively protect chickens against the same serovars as present in the vaccine, however, protection against heterologous serovars is a topic for debate (Cooper et al., 1994; Dueger et al., 2003; Gantois et al., 2006b; Matulova et al., 2013a). Moreover, due to the time necessary for the induction of protective immunity and elimination of the vaccine strain from the vaccinated chickens, the vaccination is nearly impossible to use in broilers. Therefore, cost-effective alternatives with a broad protective efficacy are being sought.

One such alternative is to provide newly hatched chickens with beneficial microbiota since the current commercial poultry production system has eliminated any contact between hens and chickens. Chicks are hatched in a clean hatchery environment and egg surface cleaning and disinfection further minimize microbiota transfer, despite the fact that gut colonization of newly hatched chickens protects against *Salmonella* infection has been known for decades (Rantala and Nurmi, 1973; Wierup et al., 1988; Schneitz et al., 1991; Hofacre et al., 2002; Kerr et al., 2013; Milbradt et al., 2014). Chickens in commercial production are therefore colonized by microbiota present in the environment. The cecum is first colonized by representatives of *Enterobacteriaceae*, which are replaced by representatives of *Lachnospiraceae* and *Ruminococcaceae* in week 2 of life. From approximately week 5 of life, representatives of phylum *Bacteroidetes* can be detected in chicken cecal microbiota and a relatively stable microbiota composition is achieved at sexual maturity around week 18 of life (Videnska et al., 2014). Despite this, the cecum of newly hatched chickens can be colonized by microbiota of nearly any composition, given this is provided to the chickens (Polansky et al., 2016). The possibility to easily colonize the cecum of newly hatched chickens by different microbiota immediately raises the question whether microbiota of different composition will similarly protect chickens against *Salmonella* infection since there may be considerable differences in the metabolome of microbial communities of a different composition.

It is commonly accepted that the production of short chain fatty acids, particularly butyrate, are critically important for energy metabolism in host epithelial cells (Fleming et al., 1991). In addition, butyrate also suppresses the expression of the cell invasion associated type III secretion system of *Salmonella* (Van Immerseel et al., 2003; Gantois et al., 2006a). Butyrate producers in the gut microbiota are commonly found in different representatives of *Firmicutes* which are under-represented in the microbiota of chickens in the first week of life, dominant in young chickens 2–4 weeks of age and form around 50% of total microbiota in adult hens (Videnska et al., 2014; Polansky et al., 2016). This is why in this study we tested whether the microbiota of different composition from hens of different age would similarly protect chickens against *Salmonella* Enteritidis (*S*. Enteritidis) infection. We tested not only the prophylactic efficacy of gut microbiota administration but also therapeutic administration of cecal microbiota and found that microbiota was highly protective against *S*. Enteritidis infection if administered prophylactically but failed to protect chickens if administered therapeutically after *S*. Enteritidis infection.

## Materials and Methods

### Ethical Statement

The handling of animals in the study was performed in accordance with current Czech legislation (Animal Protection and Welfare Act 246/1992). The specific experiments were approved by the Ethics Committee of the Veterinary Research Institute followed by the Committee for Animal Welfare of the Ministry of Agriculture of the Czech Republic (permit number MZe 1480).

### Experimental Design

In the first experiment we tested the resistance of chickens of increasing age to *S*. Enteritidis infection. Four newly hatched male ISA Brown chickens were infected with *S*. Enteritidis each on days 1, 2, 3, 4, 5, 6, 7, 9, 12, 16, 19 and 22, and sacrificed 4 days later. *S*. Enteritidis counts in the liver were determined as described below.

In the second experiment, 90 newly hatched male ISA Brown chickens were divided into six groups. Chickens in group W1 were inoculated with cecal microbiota from 1-week-old donor chickens, group W3 was inoculated with microbiota from 3-week-old chickens, group W16 was inoculated with microbiota from 16-week-old hens, group W28 was inoculated with microbiota from 28-week-old hens and group W42 was inoculated with microbiota from 42-week-old hens. The last group included 15 non-colonized chickens (NC). All the recipient chickens were inoculated with cecal extracts on day 1 of life. On day 8 of life, three chickens from each group were sacrificed to check for microbiota composition. Of the remaining 12 chickens in each group, six chickens were infected with *S*. Enteritidis. Four days later, the experiment was terminated and all chickens were sacrificed.

In the last experiment, 42 newly hatched chickens were divided into six groups. Group NC-NI included non-colonized and *S*. Enteritidis non-infected chickens. Group COL was inoculated on day 1 of life with cecal microbiota from three 35-week-old hens and remained uninfected with *S*. Enteritidis. Cecal extract from 35-week-old hens was used as that containing well-established and protective microbiota. Group INF was infected on day 1 of life with *S*. Enteritidis. Group INF+COL was infected with *S*. Enteritidis and inoculated with microbiota on day 1 of life. Group INF-COL was infected on day 1 with *S*. Enteritidis followed by inoculation with microbiota on day 2. Chickens in the group COL-INF were inoculated with microbiota on day 1 of life followed by *S*. Enteritidis infection on day 2 of life. The experiment was terminated when the chickens were 5 days old.

In all experiments, male newly hatched ISA Brown chickens were obtained from a local commercial hatchery on the day of hatching. Chickens and hens used for collecting cecal microbiota were obtained from a local commercial egg laying hen farm. To prepare cecal extracts from donors for the inoculation of recipient chickens, approximately 0.5 g cecal content was collected and resuspended in 5 ml of PBS with 0.05% L-cysteine. After 5 min decanting, a mix of equal volumes of the extracts from the donors of the same age was formed and 0.1 ml of this mix was orally applied to newly hatched chickens. All experimental infections were performed orally with 1 × 10^7^ CFU *S*. Enteritidis 147 spontaneously resistant to nalidixic acid (Methner et al., 2004) with proven virulence for chickens and mice (Rychlik et al., 2009; Karasova et al., 2010). Chickens were reared in perforated plastic boxes with free access to water and feed and each experimental or control group was kept in a separate room. Chickens were sacrificed under chloroform anesthesia and during necropsy, 0.5 g liver or cecum was collected to enumerate *S*. Enteritidis. In addition, sections of cecal tissue were collected in RNALater (Qiagen) and stored at -80°C prior RNA purification. Finally, cecal contents were collected and frozen at -20°C for microbiota characterization.

### Enumeration of *S.* Enteritidis

The samples were homogenized in peptone water, 10-fold serially diluted and plated on XLD agar supplemented with 20 μg/ml nalidixic acid. The detection limit of direct plating was 500 CFU/g of sample. Samples negative after direct plating were subjected to enrichment in modified semi-solid Rappaport-Vassiliadis medium for qualitative *S*. Enteritidis determination. Counts of *S*. Enteritidis found positive after direct plating were logarithmically transformed. Samples positive only after enrichment were assigned a value of 1 and negative samples were assigned a value of 0.

### Quantitative Reverse-Transcriptase PCR

Cecal tissue samples collected from the middle part of each cecum were recovered from RNALater, mixed with 1 ml TRI Reagent (MRC) and homogenized with a MagNALyzer (Roche). Fifty μl of 4-bromoanisole (MRC) was added to the homogenate and after centrifugation for 15 min at 14, 000 × *g*, the upper phase containing RNA was collected and purified with RNeasy Mini Kit (Qiagen). The concentration of RNA was determined spectrophotometrically (Nanodrop, Thermo Scientific) and 1 μg of RNA was immediately reverse transcribed into cDNA using M-MLV reverse transcriptase (Invitrogen) and oligo(dT) primers. Following reverse transcription, the cDNA was diluted 10× with sterile water and stored at -20°C prior to quantitative real-time PCR.

The chicken response to microbiota colonization and *S*. Enteritidis infection was characterized by determining the expression of 24 different genes. Primer sequences for the quantification of gene expression by real-time PCR have been published previously (Crhanova et al., 2011; Matulova et al., 2012, 2013b). Real-time PCR was performed in 3 μl volumes in 384-well microplates using QuantiTect SYBR Green PCR Master Mix (Qiagen) and Nanodrop II Stage pipetting station (Innovadyne) for PCR mix dispensing. Amplification and signal detection were performed using a LightCycler II (Roche) with an initial denaturation at 95°C for 15 min followed by 40 cycles of 95°C for 20 s, 60°C for 30 s and 72°C for 30 s. Each sample was subjected to real-time PCR in duplicate and the mean Ct value of the duplicates was used for subsequent calculations. The Ct values of the genes of interest were normalized (ΔCt) to an average Ct value of three house-keeping genes glyceraldehyde-3-phosphate dehydrogenase (GAPDH), TATA box binding protein (TBP), and ubiquitin (UB; Crhanova et al., 2011) and the relative expression of each gene of interest was then calculated as 2^-ΔCt^.

### Sequencing V3/V4 Region of 16S rRNA Genes

Cecal content samples were homogenized in a MagNALyzer (Roche). Following homogenization, the DNA was extracted using the QIAamp DNA Stool Mini Kit according to the manufacturer’s instructions (Qiagen). The DNA concentration was determined spectrophotometrically and DNA was stored at -20°C until use. DNA samples from the cecal contents of 54 chickens were diluted to the same concentration of 5 ng/μl and were used as a template in PCR with forward primer 5′- *TCGTCGGCAGCGTCAGATGTGTATAAGAGACAG*-MID-GT-CCTACGGGNGGCWGCAG-3′ and reverse primer 5′-*GTCTCGTGGGCTCGGAGATGTGTATAAGAGACAG*-MID-GT-GACTACHVGGGTATCTAATCC-3′. The sequences initalics served as index and adapter ligation whereas the underlined sequences allowed for the amplification over V3/V4 region of 16S rRNA genes. MIDs represent different sequences of 5, 6, 9, or 12 base pairs in length which were used to differentiate samples within the sequencing groups. PCR amplification and clean up were performed using KAPA Taq HotStart PCR Kit (Kapa Biosystems) following the protocol for 16S metagenomic sequencing library preparation recommended by Illumina. In the next step the DNA concentration was determined fluorometrically and the DNA was diluted to 100 ng/μl. Groups of 14 PCR products with different MID sequences were pooled and indexed with a Nextera XT Index Kit (Illumina). Prior to sequencing, the concentration of differently indexed samples was determined using the KAPA Library Quantification Complete kit (Kapa Biosystems). All indexed samples were diluted to 7 ng/μl and 20% of phiX DNA was added. Sequencing was performed using MiSeq Reagent Kit v3 and MiSEQ 2000 apparatus according to the manufacturer’s instructions (Illumina).

The fastq files generated as an Illumina sequencing output were uploaded into Qiime software (Caporaso et al., 2010). Reverse reads from pair end sequencing were shortened to 250 base pairs long and pair ends were joined. Quality trimming criteria were set to a value of 19 and no mismatch in the MID sequences. In the next step, chimeric sequences were predicted by slayer algorithm and excluded from the analysis. The resulting sequences were then classified with RDP Seqmatch with an operational taxonomic units (OTUs) discrimination level set to 97%.

### Statistics

*Salmonella* Enteritidis counts in the liver and cecum were compared with ANOVA followed by Tukey *post hoc* test. Heat maps and clustering used for the characterization of the chicken inflammatory response were generated in R-studio. The similarity of microbiota populations was determined by UniFrac analysis followed by principal coordinate analysis (PCoA) implemented in Qiime software.

## Results

### Resistance of Chickens of Increasing Age to *S.* Enteritidis Infection

**FIGURE 1:**
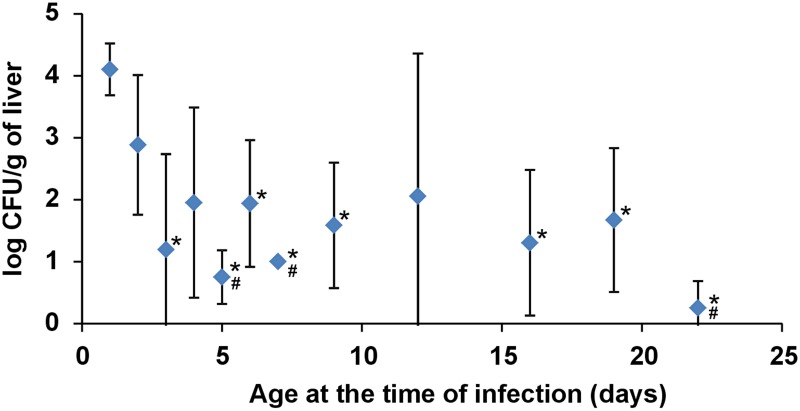
***Salmonella* counts in the liver of chickens infected with *S*. Enteritidis at different ages.** Four chickens were infected on the day of life shown on the axis X and sacrificed 4 days later. Resistance to *S*. Enteritidis rapidly increased during the first week of life but did not change from week 2 onward. * significantly different from the chickens infected on day 1; ^#^ significantly different from the chickens infected on day 2 (*P* < 0.05, ANOVA followed by Tukey test).

Chicken resistance to *S*. Enteritidis infection increased quickly during the first week of life and was sustained until the end of the experiment (last group of chickens was infected on day 22 of life, **Figure 1**).

### Protective Effect of Microbiota of Different Composition Against *S.* Enteritidis Infection

**FIGURE 2:**
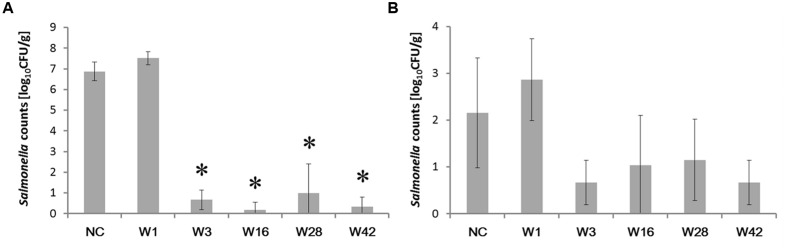
***Salmonella* counts in the cecum and liver 4 days post-infection of chickens inoculated by cecal microbiota from chickens of different age.** Chickens were inoculated with microbiota on day 1, infected with *S*. Enteritidis on day 8 and sacrificed on day 12 of life. **(A)** *S*. Enteritidis counts in the cecum. **(B)** *S*. Enteritidis counts in the liver. Data are presented as mean ± SD. NC – non-colonized but *S*. Enteritidis infected control group; W1 – *S*. Enteritidis counts in the chickens inoculated with microbiota from 1-week-old chickens; W3 – *S*. Enteritidis counts in the chickens inoculated with microbiota from 3-week-old chickens; W16 – *S*. Enteritidis counts in the chickens inoculated with microbiota from 16-week-old chickens; W28 – *S*. Enteritidis counts in the chickens inoculated with microbiota from 28-week-old hens; W42- *S*. Enteritidis counts in the chickens inoculated with microbiota from 42-week-old hens. Asterisks indicate statistically significant differences (*P* < 0.05, ANOVA followed by Tukey test) compared to the non-colonized but infected chickens.

Since *Enterobacteriaceae* are replaced with different *Firmicutes* representatives in the second week of life followed by the appearance of *Bacteroidetes* later in life (Videnska et al., 2014), we subsequently determined the protective effect of cecal microbiota from 1-, 3-, 16-, 28-, and 42-week-old hens against *S*. Enteritidis. *Salmonella* was not detected in pooled samples from cecal microbiota inoculated but *S*. Enteritidis non-infected chickens in any of the experiments. Following *S*. Enteritidis infection, *Salmonella* counts in the ceca were significantly higher in the non-colonized chickens and the chickens inoculated with microbiota from 1-week-old donors than in the chickens inoculated with microbiota from 3-week-old or older chickens. Similar results were recorded also for liver colonization, though in the liver, statistically significant protection against *S*. Enteritidis challenge was not recorded due to low *S*. Enteritidis counts and the appearance of *S*. Enteritidis negative chickens (**Figure 2**).

### Protective Effect of Microbiota of Different Composition Against the Inflammatory Response Caused by *S.* Enteritidis Infection

**FIGURE 3:**
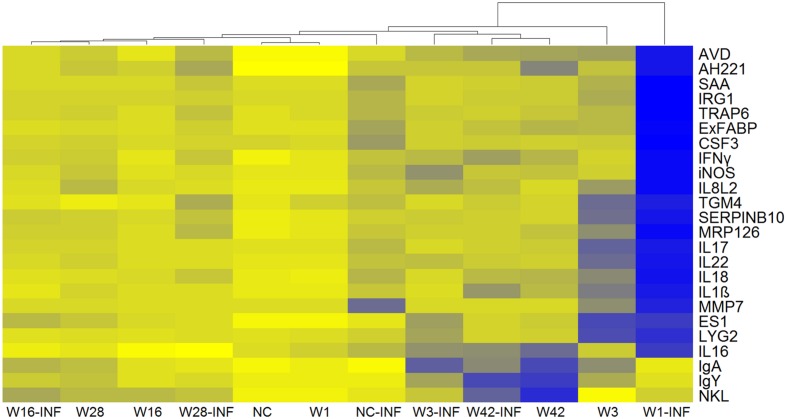
**Heat map of gene expression in the ceca of 12-day-old chickens.** Yellow, the lowest expression, blue, the highest expression. NC – Non-colonized and non-infected control group; NC-INF – Non-colonized group infected with *S*. Enteritidis; W1(-INF) – Chickens inoculated with microbiota from 1-week-old chickens (and infected with *S*. Enteritidis on day 8 of life); W3-(INF) – Chickens inoculated with microbiota from 3-week-old chickens (and infected with *S*. Enteritidis on day 8 of life); W16-(INF) – Chickens inoculated with microbiota from 16-week-old chickens (and infected with *S*. Enteritidis on day 8 of life); W28-(INF) – Chickens inoculated with microbiota from 28-week-old chickens (and infected with *S*. Enteritidis on day 8 of life); W42-(INF) – Chickens inoculated with microbiota from 42-week-old chickens (and infected with *S*. Enteritidis on day 8 of life).

Next we were interested whether inoculation with microbiota induced any inflammatory response which could explain the resistance to subsequent *S*. Enteritidis challenge and whether microbiota protected chickens from an inflammatory response to *S*. Enteritidis infection. Twelve-day-old chickens inoculated with microbiota from 1-, 16-, and 28-week-old donors did not develop any inflammation in the cecum and clustered with the non-colonized and non-infected control chickens (**Figure 3**). On the other hand, chickens responded to the inoculation with microbiota from 3- and 42-week-old donors. Microbiota from 3-week-old donors induced expression of cytokines such as IL8, IL1β, IL22, IL17, and IL18 and microbiota from 42-week-old donors induced expression of NK-lysin (NKL), IL16, IgY, and IgA (**Figure 3**).

Colonization with microbiota from 3-week-old or older chickens protected chickens from an inflammatory response to infection with *S*. Enteritidis. Non-colonized chickens responded to *S*. Enteritidis infection by a mild increase in gene expression, mostly in genes coding for acute phase proteins (e.g., SAA, AVD, TRAP, IRG1, or ExFABP), despite high *S*. Enteritidis counts in the cecum (compare **Figures 2** and **3**). Chickens inoculated with microbiota from 1-week-old donors were the most sensitive to *S*. Enteritidis and, except for NKL, IgY and IgA, responded to *S*. Enteritidis challenge by a high induction of all tested genes (**Figure 3**). Microbiota from adult hens therefore reduced colonization of chickens after *S*. Enteritidis challenge, although certain microbiota compositions may even sensitize the inoculated chickens to *S*. Enteritidis challenge.

### Composition of Cecal Microbiota in the Inoculated Chickens

**FIGURE 4:**
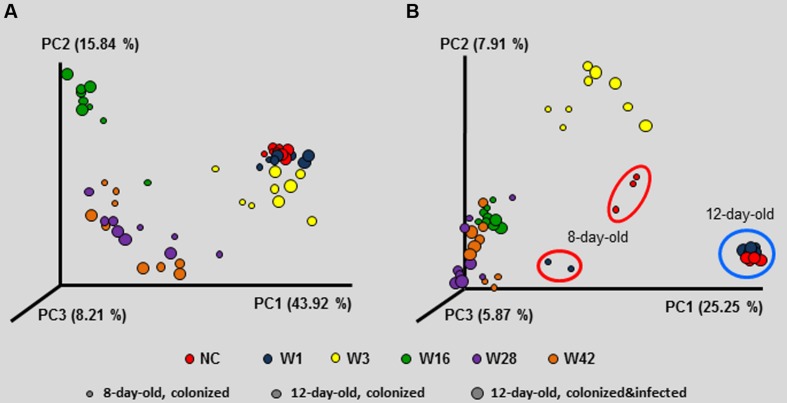
**Principal coordinate analysis (PCoA) characterizing the microbiota composition of 8- and 12-day-old chickens colonized on day 1 of life with cecal microbiota from donor chickens of different age, as well as 12-day-old chickens inoculated with microbiota from different donors on day 1, challenged with *S*. Enteritidis on day 8 and sacrificed on day 12 of life.** Composition of microbiota in the inoculum was the most decisive factor which dominated over age when the inoculated chickens were sacrificed and *S*. Enteritidis infection status, both in weighted PCoA **(A)** and unweighted PCoA **(B)**. Unweighted PCoA showed that although non-colonized 12-day-old chickens exhibited similar microbiota as the chickens inoculated with microbiota from 1-week-old donors, there was different microbiota composition on day 8, i.e., at the time of *S*. Enteritidis infection which may explain the different inflammatory response shown in **Figure 3**.

Since the inoculation with microbiota affected resistance to *S*. Enteritidis challenge, next we determined the microbiota composition in the resistant and sensitive chickens, before and after the infection. The inoculum itself (and therefore the microbiota present in the donor chickens, though we did not determine this specifically in this study) was the most decisive factor for the composition of cecal microbiota in the recipient chickens since the chickens inoculated with the cecal extract from different donor chickens formed separate clusters. These clusters always comprised 8 and 12-day-old chickens irrespective of *S*. Enteritidis infection (**Figure 4**). The only exception was the clustering of 8-day-old non-colonized chickens and the chickens inoculated with microbiota from 1-week-old donors on day 8 of life, i.e., prior to infection with *S*. Enteritidis. These chickens formed separate clusters using unweighted PCoA analysis (**Figure 4**).

### Identification of Protective Microbiota Members

Unweighted PCoA analysis indicated that there must have been different OTUs in 8-day-old non-colonized chickens and chickens inoculated with microbiota from 1-week-old donors and these differences could be responsible for the different inflammatory response after *S*. Enteritidis infection. There were 72 OTUs which were present in the microbiota of all three non-colonized chickens on day 8 of life and were absent in microbiota of two chickens inoculated with microbiota from 1-week-old donors (unfortunately we failed with analysis of one chicken in this group). However, only three of these OTUs formed more than 0.05% of total microbiota and these were assigned to genera *Alistipes*, *Clostridium* XIVa, and *Blautia*. On the other hand, there were 130 OTUs specifically present in the chickens inoculated with cecal extracts from 1-week-old donors and 11 of these formed more than 0.05% of total microbiota. These OTUs belonged to genera *Pediococcus* (two different OTUs), *Bacteroides* (two different OTUs), *Clostridium* IV, *Clostridium* XVIII, *Faecalibacterium*, *Streptophyta*, and unclassified members of families *Ruminococcaceae*, *Lachnospiraceae*, and *Prevotellaceae*. The most dominant were the two *Pediococci* OTUs which together formed 1.7% of all microbiota in the chickens inoculated with microbiota from 1-week-old donors.

### Therapeutic Use of Microbiota Administration

In the last experiment we tested whether the protective effect can be achieved earlier than 7 days after inoculation and whether the microbiota inoculation can be used therapeutically. Newly hatched chickens were therefore inoculated with microbiota on the day of hatching and challenged with *S*. Enteritidis 24 h later, or microbiota and *S*. Enteritidis were administered simultaneously on day 1 of life, or the chickens were infected with *S*. Enteritidis on the day of hatching and microbiota were provided to the chickens therapeutically 24 h later.

**FIGURE 5:**
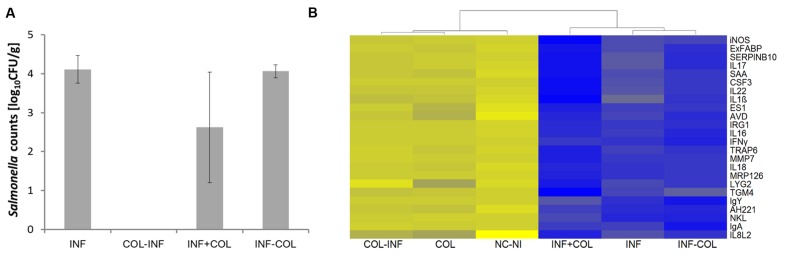
**Preventive and therapeutic use of chicken gut microbiota for the reduction of *S*. Enteritidis colonization. (A)** *Salmonella* counts in the liver 4 days post-infection (DPI). INF – Chickens infected on day 1 of life; COL-INF – Chickens colonized on day 1 of life and infected on day 2 of life; INF+COL- Chickens infected and colonized on day 1 of life; INF-COL – Chickens infected on day 1 of life and colonized on day 2 of life. Data are presented as mean ± SD. **(B)** Heat map of gene expression in the chicken cecum on day 5 of life; yellow, low expression, blue, high expression. COL-INF – Chickens colonized on day 1 of life with 35-week-old microbiota and infected with *S*. Enteritidis on day 2 of life; COL – Chickens colonized with 35-week-old microbiota; NC-NI – Control group of chicken, non-colonized and non-infected; INF+COL – Chickens infected and colonized on day 1 of life; INF – Chickens infected with *S*. Enteritidis; INF-COL – Chickens infected on day 1 of life and colonized on day 2 of life.

No *S*. Enteritidis was detected in the liver of chickens which were colonized with microbiota on the day of hatching, infected with *S*. Enteritidis 24 h later and sacrificed on day 5 of life (**Figure 5A**). In all other combinations, i.e., in the non-colonized but *S*. Enteritidis infected chickens, in the *S*. Enteritidis infected and 24 h later microbiota inoculated chickens, or in the chickens simultaneously inoculated with microbiota and infected with *S*. Enteritidis, *S*. Enteritidis could be detected in the liver on day 5 of life, though a partial protective effect of administered microbiota was recorded in the chickens in which microbiota was administered simultaneously with *S*. Enteritidis (**Figure 5A**).

Quantitative real-time PCR performed with mRNA/cDNA purified from cecal tissue confirmed the results from liver colonization. Inoculation with microbiota had only a minimal effect on the cecal inflammatory response and also subsequent infection with *S*. Enteritidis did not result in an inflammatory response. On the other hand, when the chickens were infected with *S*. Enteritidis before or together with cecal microbiota inoculation, there was an extensive inflammatory response similar to that observed in the non-colonized but *S*. Enteritidis infected chickens (**Figure 5B**). Administration of microbiota therefore was not of any therapeutic effect.

## Discussion

Though, it has been repeatedly reported that the resistance of chickens to infection with non-typhoid serovars of *Salmonella* increases with age, such experiments were performed with chickens aged weeks or months (Beal et al., 2004, 2005; Withanage et al., 2004) and data on the development of resistance in the early days of life are less common (Crhanova et al., 2011). In this study we therefore first tested how resistance increases within the first days of life and observed that the increase in resistance is a matter of days. We reported earlier that around day 4 of life, a mild inflammatory signaling can be recorded (Crhanova et al., 2011) which may serve as a signal for the infiltration of leukocytes to the cecal mucosa (Van Immerseel et al., 2002; Bar-Shira et al., 2003) and increase in resistance to *Salmonella* infection. Though inflammation and leukocyte infiltration likely contributes to chicken resistance to *Salmonella*, we consider the presence of microbiota itself as a more important factor since we were not able to recover *S*. Enteritidis following microbiota pre-inoculation at all. Microbiota, if administered prior *S*. Enteritidis infection, therefore directly restricted *S*. Enteritidis multiplication and the absence of inflammation was only a consequence of the absence of *S*. Enteritidis in the cecum.

Interestingly, microbiota from 1-week-old chickens did not protect chicken recipients against *S*. Enteritidis infection. Though not reaching statistical significance, *S*. Enteritidis counts were slightly higher in the chickens inoculated with microbiota from 1-week-old chickens than in the non-colonized controls (**Figure 2**). This minor difference was, however, enough to result in a significantly different inflammatory response in these two groups of chickens (**Figure 3**). In other words, chickens that received cecal microbiota from 1-week-old donors were sensitized to the inflammatory response to *S*. Enteritidis infection. Microbiota analysis showed that prior to the infection, the cecal microbiota differed in the non-colonized controls and chickens inoculated with microbiota from 1-week-old donors. However, it should be reminded that the microbiota of very young chickens is quickly developing (Videnska et al., 2014) and under different rearing conditions, the protective microbiota may appear slightly earlier or later during life. It is also difficult to speculate which of the microbiota members present in significantly higher abundance in the inoculated chickens was responsible for their higher sensitivity to *S*. Enteritidis infection. Gram positive *Pediococcus* or *Faecalibacterium* were present in the microbiota of the inoculated chickens at higher abundance than in the non-colonized controls but these bacteria are used either as probiotics or were reported as having an anti-inflammatory effect (Sokol et al., 2008; Biloni et al., 2013; Qiu et al., 2013). These two bacterial species were therefore quite unlikely to be responsible for the increased sensitivity to *S*. Enteritidis. On the other hand, our unpublished results show that inoculation of newly hatched chickens with pure cultures of certain clones belonging to genera *Bacteroides* sp. and *Alistipes* sp. results in a worse course of infection following *S*. Enteritidis challenge than in the control, non-colonized chickens. The two different clones of *Bacteroides* sp. present in the inoculated chickens may therefore explain their increased sensitivity to *S*. Enteritidis, though, of course, this cannot be considered as a definitive conclusion.

It is not surprising that such results were not reported earlier since all studies testing competitive exclusion used adult hens as donors of microbiota (Kerr et al., 2013; Milbradt et al., 2014) and nobody tested microbiota from 1-week-old chickens. Moreover, microbiota in 1-week-old chickens may fluctuate and the (non)protective effect may vary from experiment to experiment. Despite this, these results show that care must be taken concerning the composition of microbiota used for competitive exclusion since some may not provide the expected protection or may allow for overgrowth of opportunistic pathogens or zoonotic agents in the ceca of inoculated chickens (Polansky et al., 2016).

The protective effect of microbiota inoculation could be achieved within 24 h further supporting the hypothesis that this effect is independent of the chicken immune system. Unfortunately, therapeutic administration was not successful and even simultaneous administration of *S*. Enteritidis and cecal microbiota did not prevent *S*. Enteritidis colonization. However, we should note that the infectious dose of *S*. Enteritidis in these experiments was deliberately high, higher than in field conditions. The microbiota administration in field conditions may therefore increase chicken resistance to *S*. Enteritidis even if being administered in parallel to *Salmonella* natural infection. In addition, the administration of microbiota in field conditions may protect non-infected chickens thus decreasing *S*. Enteritidis spread on a flock level, despite the fact that competitive exclusion products work better under experimental conditions than in field trails. Interestingly, since the chicken cecal immune system responded to *S*. Enteritidis infection during co-administration with microbiota, it should equally respond and recognize attenuated *Salmonella* vaccine strains if administered together with microbiota, as reported earlier (Methner et al., 1999). This also means that it might be possible to develop a vaccine which would consist of an attenuated *Salmonella* strain and selected microbiota. Microbiota will protect chickens during the early days of life followed by specific long term antigen specific protection due to the vaccination.

## Author Contributions

KV and IR designed the experiments, analyzed data and wrote the manuscript. TK and LD-G characterized gut microbiota composition. AS and MF performed real time PCR. FS and HH were responsible for animal handling and sample collection.

## Conflict of Interest Statement

The authors declare that the research was conducted in the absence of any commercial or financial relationships that could be construed as a potential conflict of interest.
